# Differential amplification of satellite *PaB6* in chromosomally hypervariable *Prospero autumnale* complex (Hyacinthaceae)

**DOI:** 10.1093/aob/mcu178

**Published:** 2014-08-28

**Authors:** Khatere Emadzade, Tae-Soo Jang, Jiří Macas, Ales Kovařík, Petr Novák, John Parker, Hanna Weiss-Schneeweiss

**Affiliations:** 1 Department of Botany and Biodiversity Research, University of Vienna, Rennweg 14, A-1030 Vienna, Austria; 2 Czech Academy of Sciences, Institute of Plant Molecular Biology, Ceske Budejovice, Czech Republic; 3 Czech Academy of Sciences, Institute of Biophysics, Brno, Czech Republic; 4 Cambridge University Botanic Garden, Cambridge CB2 1JF, UK

**Keywords:** *PaB6*, *Prospero autumnale*, Hyacinthaceae, chromosomal evolution, copy number, differential amplification, fluorescence *in situ* hybridization (FISH), genome size, pericentric satellite DNA, next-generation sequencing

## Abstract

**Background and Aims:**

Chromosomal evolution, including numerical and structural changes, is a major force in plant diversification and speciation. This study addresses genomic changes associated with the extensive chromosomal variation of the Mediterranean *Prospero autumnale* complex (Hyacinthaceae), which includes four diploid cytotypes each with a unique combination of chromosome number (*x* = 5, 6, 7), rDNA loci and genome size.

**Methods:**

A new satellite repeat *PaB6* has previously been identified, and monomers were reconstructed from next-generation sequencing (NGS) data of *P. autumnale* cytotype B^6^B^6^ (2*n* = 12). Monomers of all other *Prospero* cytotypes and species were sequenced to check for lineage-specific mutations. Copy number, restriction patterns and methylation levels of *PaB6* were analysed using Southern blotting. *PaB6* was localized on chromosomes using fluorescence *in situ* hybridization (FISH).

**Key Results:**

The monomer of *PaB6* is 249 bp long, contains several intact and truncated vertebrate-type telomeric repeats and is highly methylated. *PaB6* is exceptional because of its high copy number and unprecedented variation among diploid cytotypes, ranging from 10^4^ to 10^6^ copies per 1C. *PaB6* is always located in pericentromeric regions of several to all chromosomes. Additionally, two lineages of cytotype B^7^B^7^ (*x* = 7), possessing either a single or duplicated 5S rDNA locus, differ in *PaB6* copy number; the ancestral condition of a single locus is associated with higher *PaB6* copy numbers.

**Conclusions:**

Although present in all *Prospero* species, *PaB6* has undergone differential amplification only in chromosomally variable *P. autumnale*, particularly in cytotypes B^6^B^6^ and B^5^B^5^. These arose via independent chromosomal fusions from *x* = 7 to *x* = 6 and 5, respectively, accompanied by genome size increases. The copy numbers of satellite DNA *PaB6* are among the highest in angiosperms, and changes of *PaB6* are exceptionally dynamic in this group of closely related cytotypes of a single species. The evolution of the *PaB6* copy numbers is discussed, and it is suggested that *PaB6* represents a recent and highly dynamic system originating from a small pool of ancestral repeats.

## INTRODUCTION

Genomes of higher plants contain a spectrum of repetitive DNAs (Schmidt and Heslop-Harrison, 1998; Macas *et al*., 2002; Ugarković and Plohl, 2002; Hemleben *et al*., 2007). This repetitive fraction is predominantly composed of dispersed mobile genetic elements (DNA transposons, retroelements) and tandemly repeated satellite DNAs (Hemleben *et al*., 2007; Weiss-Schneeweiss and Schneeweiss, 2013). Satellite DNA is typically species or genus specific, consisting of long arrays of late-replicating, tandemly arranged, head-to-tail repeats (Charlesworth *et al*., 1994; Richard *et al*., 2008).

Satellite DNA is a non-coding fraction of the genome of limited transcriptional capacity, subject to methylation, histone modification and chromatin remodelling (Volkov *et al*., 2006; Hemleben *et al*., 2007). It is preferentially localized in heterochromatic pericentromeric and sub-telomeric chromosomal regions, but also occurs interstitially (Charlesworth *et al*., 1994; Hemleben *et al*., 2007). No general function has been ascribed to satellite DNA (Ugarković and Plohl, 2002; Hemleben *et al*., 2007), although biological roles have been suggested for its specific families – the maintenance of chromosome structure (Ferree and Prasad, 2012), recognition of homologous chromosomes during meiosis (Willard, 1998; Ferree and Prasad, 2012), regulation of gene expression (Pezer *et al*., 2012), and heterochromatin organization and centromere function (Csink and Henikoff, 1998; Ugarković and Plohl, 2002; Ugarković, 2005; Hemleben *et al*., 2007; Martins *et al*., 2008; Gong *et al*., 2012; Pezer *et al*., 2012).

Higher plant genomes have from a few to many families of satellite DNAs (Hemleben *et al*., 2007; Macas *et al*., 2007, 2011). Individual satellite DNA families in a genome differ in sequence and copy number. Thus, one or a few families are usually present in high copy number, while others have low numbers of repeats (Hemleben *et al*., 2007). It has been proposed that groups of related taxa share a common ‘library’ of satellite DNA families, each of which may follow its own evolutionary trajectory (Meštrovič *et al*., 1998). As species diverge, some satellite DNA families reduce in copy number, or even disappear, while others amplify, and new variants may arise (Meštrovič *et al*., 1998; Nijman and Lenstra, 2001; Pons *et al*., 2004). Newly arising variants of a satellite DNA can rapidly replace previous copies due to concerted evolution, which results in intraspecific sequence homogenization (Plohl, 2010). The efficiency of homogenization is satellite DNA specific and depends on initial copy number, genomic location, repeat length and mode of reproduction (Dover, 1982; Stephan and Cho, 1994; Plohl *et al*., 2008; Navajas-Pérez *et al*., 2009; Kuhn *et al*., 2010). All these changes may parallel, or even precede, species diversification (Elder and Turner, 1995; Koukalova *et al*., 2010; Raskina *et al*., 2011; Belyayev and Raskina, 2013). Plant satellite DNA families are often derived from fragments of standard components of the genome, such as 35S rDNA (Lim *et al*., 2004; Almeida *et al*., 2012), 5S rDNA (Vittorazzi *et al*., 2011) or transposable elements (Sharma *et al*., 2013). Their subsequent evolution involves various processes such as replication slippage, unequal crossing-over, gene conversion or extrachromosomal circular DNA (eccDNA) formation (Smith, 1976; Walsh, 1987; Charlesworth *et al*., 1994; Elder and Turner, 1995; Cohen *et al*., 2008; Navrátilová *et al*., 2008).

The genus *Prospero* (Hyacinthaceae) consists of two chromosomally and morphologically stable species, *P. hanburyi*, 2*n* = 14 and *P. obtusifolium*, 2*n* = 8, and a chromosomally variable species complex referred to as *P. autumnale. Prospero autumnale* consists of a spectacular, and unparalleled, array of genetically, chromosomally and phylogenetically well-defined, recently evolved, diploid cytotypes, and a large array of polyploid derivatives (Vaughan *et al*., 1997; Jang *et al*., 2013). This complex shows near homogeneity in its morphology, and provides an excellent system for comparative and evolutionary genomic studies. It is distributed across the whole Mediterranean basin (Speta, 1998; Jang *et al*., 2013). Four chromosomally distinct diploid lineages (cytotypes) have been described*,* each of which possesses a unique combination of basic chromosome number (*x* = 5, 6, 7), DNA content and localization of rDNAs (Vaughan *et al*., 1997; Jang *et al*., 2013). Two cytotypes based on *x* = 7 are referred to as B^7^B^7^, distributed across the whole Mediterranean basin, and AA, which has larger chromosomes and genome size and is confined to the western-most Mediterranean and the Atlantic coast of Morocco, Portugal and Spain. The other two diploid cytotypes – with 2*n* = 12 (B^6^B^6^) and 2*n* = 10 (B^5^B^5^) – originated from a putative ancestor with 2*n* = 14 via independent chromosome fusions. B^6^B^6^ is endemic to Crete while B^5^B^5^ is endemic to Libya. With the exception of the most recently evolved cytotype B^5^B^5^, all diploids hybridize and undergo polyploidization in nature to give auto- and allopolyploids. Amongst polyploids, tetraploid and hexaploid cytotypes are most common and widespread (Ainsworth *et al*., 1983; Vaughan *et al*., 1997).

Phylogenetic and evolutionary relationships of the three species of *Prospero* have recently been established, and the ancestral basic number for the *P. autumnale* complex was inferred to be *x* = 7 (Jang *et al*., 2013). Evolution of the cytotypes AA and B^6^B^6^ has been shown to be accompanied by independent genome size increases (Jang *et al*., 2013). Large heterochromatic blocks, however, have been detected only in cytotype B^6^B^6^ (Ebert *et al.*, 1996).

Recent developments in high-throughput next-generation sequencing (NGS; Margulies *et al*., 2005) allow in-depth analyses of all components of any genome (Wicker *et al*., 2009; Deschamps and Campbell, 2010), and thus rapid identification of satellite DNAs (Macas *et al*., 2007; Torres *et al*., 2011; Heckmann *et al*., 2013). The current study involves comparative evolutionary analysis of a satellite *PaB6* identified by NGS from cytotype B^6^B^6^. Specifically, the aims are to: (1) isolate, characterize, and determine the abundance and localization of *PaB6* in the diploid species and cytotypes of *Prospero*, and their homoploid diploid hybrids; (2) assess intra- and interspecific variation of the reconstructed *PaB6* monomer at all levels of its organization – its DNA sequence, chromosomal localization and genomic abundance; (3) analyse, in a phylogenetic context, the evolutionary trajectories of *PaB6* in all six diploid cytotypes of *P. autumnale* and their diploid homoploid hybrids; and (4) discuss the dynamics of *PaB6* evolution in the context of major chromosomal rearrangements in the genus.

## MATERIALS AND METHODS

### Plant material and DNA isolation

Plants from collections of F. Speta, Linz, and J. S. Parker, Cambridge, were grown in the Botanical Garden of the University of Vienna. The plants studied and their collection details are listed in Supplementary Data Table S1. Due to the high levels of chromosomal variation in *Prospero* (Jang *et al*., 2013), every plant was karyotyped prior to analysis. Only ‘standard’ individuals without structural chromosomal variants were used.

Total genomic DNA was isolated from leaves, of several individuals each, of *P. obtusifolium*, *P. hanburyi* and the four diploid cytotypes of *P. autumnale*, including homoploid diploid hybrids (Supplementary Data Table S1) using a modified cetyltrimethylammonium bromide (CTAB) method (Doyle and Doyle, 1987; Jang *et al*., 2013).

### Next-generation sequencing and clustering-based repeat identification

Sequencing of randomly sheared total genomic DNA of the cytotype B^6^B^6^ of *P. autumnale* was performed by the Center for Medical Research, Graz, Austria using a Roche/454 GS FLX instrument with Titanium reagents (Roche Diagnostics). Sequencing half a 70 × 75 picotitre plate yielded 555 480 reads of average length 350 bp. Quality-filtered reads (397 694 corresponding to 2·2 % coverage of the genome) were subjected to graph-based clustering analysis, as described by Novák *et al*. (2010), to identify groups of reads representing repetitive elements (H. Weiss-Schneeweiss *et al*., unpubl. res.). One hundred and ninety-five out of a total of 19 751 clusters, corresponding to the most abundant families of genomic repeats, were analysed for their similarity to known sequences using RepeatMasker Open-3·0 (http://www.repeatmasker.org) and BLAST (Altschul *et al*., 1990) searches against GenBank databases and a database of plant mobile element protein sequences (Novák *et al*., 2013). Graphical layouts of individual clusters were examined using the SeqGrapheR program (Novák *et al*., 2010).

### Characterization of monomers of satellite repeats

Only one genomically abundant cluster (CL0009) was identified amongst all clusters as containing a potential satellite repeat. Structural features of the tandem repeat motif and its sub-repeats within the contigs of this cluster were further analysed with DOTTER (Sonnhammer and Durbin, 1995). Identification of the most conserved sequence variants and consensus monomer reconstruction of satellite repeat *PaB6* were conducted using *k*-mer frequency analysis as described previously (Macas *et al*., 2010), using 25 bp long *k*-mers for final sequence reconstruction.

### PCR amplification, cloning, sequencing and phylogenetic analysis of *PaB6*

**Fig. 1.**
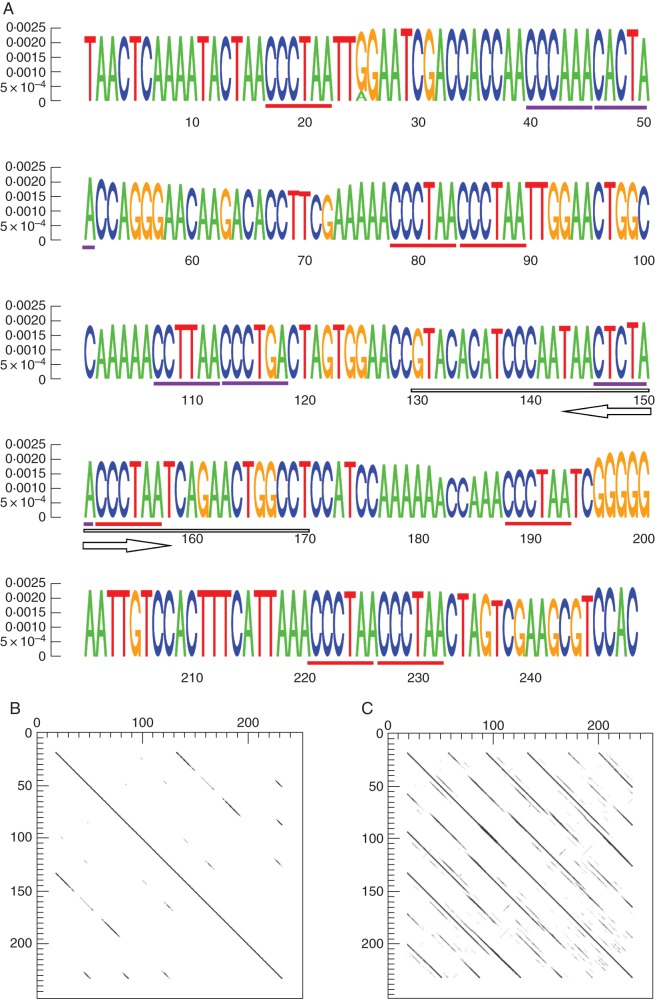
*PaB6* monomer characterization. (A) Monomer sequence logo (Schneider and Stephens, 1990) with the height of the letters corresponding to *k*-mer frequencies. Arrows indicate the origin and direction of forward and reverse primers (underlined). Perfect telomeric sequences are underlined in red, and imperfect variants in violet. (B, C) Dot plots of the monomer sequence against itself with lower (B) and higher similarity stringency (C).

**Fig. 2.**
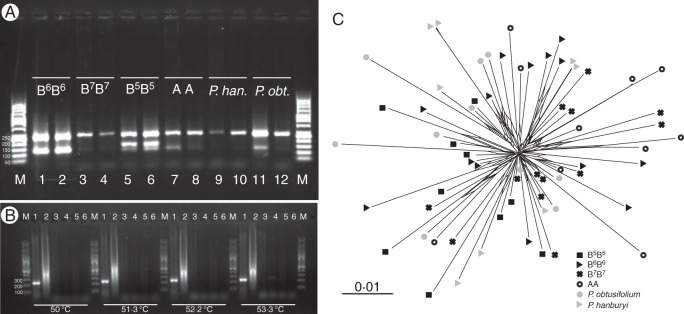
Patterns of PCR amplification of *PaB6* satellite DNA in *Prospero* and comparative phylogenetic analysis of the major monomer sequence. (A) PCR amplification products of *PaB6* monomers [M, marker; 1–2, B^6^B^6^ (H166, H427); 3–4, B^7^B^7^ (H424, H428); 5–6, B^5^B^5^ (H582, H640); 7–8, AA (H541, H550); 9–10, *P. hanburyi* (H397, H115); 11–12, *P. obtusifolium* (H559; H563; Supplementary Data Table S1)]. (B) Gradient PCR amplification of *PaB6* monomers in selected *Prospero* samples and outgroup taxa [M, marker; 1, *P. obtusifolium* H559; 2, *P. autumnale* B^6^B^6^ H166; 3, *Othocallis siberica* 2159/1; 4, *Othocallis mischtschenkoana* LI778; 5, *Barnardia scilloides* (JANG_1); 6, water as negative control] using annealing temperatures of 50–53·3 °C, as indicated. (C) Neighbour-net of *PaB6* repeats cloned from diploid cytotypes of *P. autumnale* (AA, open circles; B^5^B^5^, black filled squares; B^6^B^6^, black filled triangles; B^7^B^7^, black crosses), *P. obtusifolium* (grey circles) and *P. hanburyi* (grey triangles).

The reconstructed consensus sequence of the monomer of *PaB6* was used for the design of oligonucleotide primers (*PaB6*F, 5′-ACCCTAATCAGAACTGGCCT; *PaB6*R, 5′- TAGAGTTATTGGGATGTGTAC) facing outwards (Fig. 1A). These primers were used for amplification of *PaB6* monomers from genomic DNA of diploid *Prospero* species and cytotypes and three outgroup species (all of family Hyacinthaceae; Supplementary Data Table S1). Polymerase chain reactions consisted of 1 × buffer (MBI Fermentas, St Leon-Rot, Germany), 2·5 mm MgCl_2_ (MBI Fermentas) 0·5 μm of each of the dNTPs (MBI Fermentas), 0·2 μM of each primer (Sigma Aldrich, Vienna, Austria) and 1 U of RedTaq polymerase (Sigma Aldrich). Amplification was performed on an ABI thermal cycler 9700 (Applied Biosystems, Foster City, CA, USA) with the initial 3 min at 94 °C followed by 25 cycles each of 45 s at 94 °C, 45 s at 55 °C and 40 s at 72 °C, and a final elongation step at 72 °C for 10 min. Amplified fragments were separated on a 1·5 % agarose gel, and PCR products corresponding to the length of the monomers of satellite DNA *PaB6* were purified from the gel using Invisorb^®^ Fragment clean up (Invitek, Berlin, Germany). DNA was cloned using the pGEM-T Easy vector system and JM109 competent cells (Promega, Madison, WI, USA) following the manufacturer's instructions. Five inserts per individual were amplified from plasmids using colony PCR with universal M13 primers whereby recombinant colonies were added directly into the PCR mix and inserts amplified using reagents and conditions described in Park *et al*. (2007). Amplification products were treated with exonuclease I (ExoI) and calf intestine alkaline phosphatase (CIAP) according to the manufacturer's protocol (MBI Fermentas), and amplicons were cycle sequenced using Big Dye terminator chemistry (Applied Biosystems) and run on a 48 capillary ABI 3730 DNA Analyzer (Applied Biosystems). The sequences of satellite DNA were manually aligned in BioEdit v.7.0.9 (Hall, 1999). Phylogenetic analyses were performed using Splits-Tree (version 4.11.3; Huson and Bryant, 2006). Sequenced clones are available from GenBank under accession nos KF897587–KF897652 (Supplementary Data Table S1). Gradient PCR was performed on a peqstar thermocycler (peqlab, Erlangen, Germany) to check for the presence of *PaB6* in related genera. Primers, the PCR set-up and the PCR program used were the same as described above, except that the annealing temperatures ranged from 50 to 55 °C (Fig. 2, and not shown).

### Southern and slot blot hybridization

Abundance and restriction patterns of *PaB6* monomers in selected individuals were analysed using the Southern blot technique. A 1 μg aliquot of total genomic DNA of each *Prospero* species and cytotype was digested with 0·7 μL of *Bst*NI restriction endonuclease for 2 h at 37 °C. Digested DNA fragments were separated on a 1 % (w/v) agarose gel and transferred onto a positively charged nylon membrane, Hybond-XL, by the capillary flow method.

The probe used for hybridization was a 249 bp PCR product representing the *PaB6* satellite of *P. autumnale* cytotype B^6^B^6^ (clone 4 of individual H195; GenBank accession no. KF897620). The probe was labelled either radioactively with ^32^P (DekaLabel kit, MBI Fermentas, Vilnius, Lithuania) or using a DIG-nick translation kit (Roche, Vienna, Austria). Radioactively labelled probe was hybridized to the membrane and washed under high-stringency conditions, as described in Matyášek *et al*. (2011). Hybridization bands were visualized with a PhosphorImager (Storm, Molecular Dynamics, Sunnyvale, CA, USA), and the data were processed in ImageQuant software (Molecular Dynamics).

Hybridization of digoxigenin-labelled probe (Dig Easy Hyb, Roche, Germany) to genomic DNA was carried out at 43 °C for 14 h, and it was then washed twice in 2 × SSC (saline-sodium citrate buffer) containing 0·1 % SDS (sodium dodecylsulphate) for 5 min at room temperature, and twice in 0·5 × SSC containing 0·1 % SDS for 15 min at 65 °C. Probe was detected with CSPD chemiluminescent substrate (Roche Applied Science, USA) using Dig Wash and Block Buffer Set (Roche Applied Science, Germany), and the hybridization signals were visualized on Fusion FX7 Advance (peqlab). Due to the lower sensitivity of chemiluminescent detection compared with radioactive systems, an additional hybridization experiment was performed with cytotypes B^7^B^7^, which had been shown to possess lower amounts of satellite DNA, using 1 μg and additionally also 3 μg of genomic DNA.

The copy number of *PaB6* in all species and cytotypes was estimated using the slot blot technique. Briefly, the DNA concentration was estimated using Nanodrop 3300 (peqlab) with PicoGreen (Invitrogen) as DNA stain. Two or three dilutions of genomic DNA (100, 20 and 2 ng for B^6^B^6^ and B^5^B^5^ cytotypes; 2000, 200 and 20 ng for B^7^B^7^ and AA cytotypes; 2000 and 200 ng for *P. hanburyi* and *P. obtusifolium*), together with a series of dilutions of the unlabelled *PaB6* insert corresponding to the monomer sequence, were denatured in 0·4 m NaOH and neutralized with 0·75 m NH_4_OAc. Samples were blotted onto a positively charged Nylon membrane (peqlab) using a vacuum slot blotter (VWR, Vienna, Austria). The probe and the hybridization conditions used were the same as described above for non-radioactive Southern hybridization. Copy number was estimated using Fusion FX7 Advance software (peqlab).

### Methylation levels

The methylation level of *PaB6* repeats in the B^6^ genome was assessed using a radioactive Southern blot (see above). The genomic DNA was digested with two restriction enzymes – *Bst*NI (CCWGG) and *Scr*FI (CCNGG) – which recognize and cut nearly the same sequence, with *Scr*FI being sensitive to the inner C methylation.

### Fluorescence *in situ* hybridization

Chromosomes were prepared by enzymatic digestion and squashing (Jang *et al*., 2013). Fluorescence *in situ* hybridization (FISH), probe labelling and detection were carried out according to the method of Jang *et al*. (2013).

The probes used for FISH were a monomer of satellite DNA *PaB6* from the B^6^ genome in plasmid pGEM-T Easy and the genic region of 5S rDNA from *Melampodium montanum* (Asteraceae) in plasmid pGEM-T Easy, directly labelled with biotin or digoxigenin (Roche, Austria) by PCR (Jang *et al*., 2013). A 35S rDNA probe labelled with digoxigenin via nick translation (DIG-nick translation kit; Roche) was used in one experiment as a control for the *PaB6* probe. Digoxigenin was detected with anti-digoxigenin conjugated with fluorescein isothiocyanate (FITC; 5 μg mL^–1^; Roche) and biotin with ExtrAvidin conjugated with Cy3 (2 μg mL^–1^; Sigma Aldrich), respectively.

Commercially available, directly Cy3-labelled, PNA (peptide nucleic acid) probe to vertebrate telomeric sequences (CCCTAA)_3_ was used as the third probe, as described in the manufacturer's protocol (Telomere PNA FISH Kit/Cy3; Dako, Denmark). For the directly labelled PNA probe, after stringent washes in 2 × SSC, 0·1 × SSC and 2 × SSC with 0·2 % Tween-20 at 42 °C, for 5 min each, preparations were mounted in antifade buffer Vectashield (Vector Laboratories, Peterborough, UK) containing 4′,6-diamidino-2-phenylindole (DAPI) counterstain (2 μg mL^–1^), and stored at 4 °C.

Preparations were analysed with an AxioImager M2 epifluorescent microscope (Carl Zeiss, Vienna, Austria); images were acquired with a CCD camera, and processed using AxioVision ver. 4.8 (Carl Zeiss) with only those functions that apply equally to all pixels. At least 30 well-spread metaphases and prometaphases were analysed in each individual.

## RESULTS

### Satellite DNA identification and characterization of the monomers

Clustering analysis of the shotgun Roche/454 reads of *Prospero autumnale* cytotype B^6^B^6^ (2*n* = 12) produced thousands of clusters differing in size, corresponding to the sequence composition and genomic abundance of the various genomic repeats. A set of 195 of the largest clusters, representing the most abundant repetitive elements with genome proportions exceeding 0·01 %, was searched for features typical of satellite repeats. Only one such cluster was identified, based on the shape of the cluster graph (Novák *et al*., 2010) and the presence of tandem repeats in assembled contigs (Fig. 1; Supplementary Data Fig. S1). This novel satellite has been designated as *PaB6* – satellite DNA isolated from *P. autumnale* (*Pa*) cytotype B^6^B^6^ (*B6*). The number of reads in the cluster was 8461, or 1·8 % of the total, giving an estimate of the proportion of *PaB6* in the genome. The consensus sequence reconstruction using 25 bp long *k*-mers (Macas *et al*., 2010) resulted in a monomer of 249 bp in length (Fig. 1A), with a GC content of 44 %. Detailed analysis, using the NGS dataset, revealed two large truncated sub-repeats which could have given rise to the present-day higher order monomer of 249 bp (Fig. 1B). Each of the two sub-repeats is typically composed of three even smaller secondary sub-repeats (Fig. 1C). The complex structure of this monomer is also indicated by the pattern of *PaB6* amplification using PCR (see below).

The monomer of *PaB6* contains seven intact vertebrate-type telomeric repeats (TTAGGG) dispersed amongst other sequences and in two instances forming dimers (Fig. 1A). Additionally, five imperfect telomeric-like repeats have been identified, and potentially other repeats degenerated to a higher degree (Fig. 1A). A pentanucleotide CAAAA, conserved in many satellites (Macas *et al*., 2002), occurred three times on the top strand. In addition, there were four A4 tracts important for DNA conformation and chromatin folding (Plohl *et al*., 2010).

### Comparative sequence analysis of the monomers

The PCR amplification of the major type of the monomer, using primers designed for the reconstructed B^6^ genome monomer, resulted in products of the expected length in all four diploid cytotypes of *P. autumnale* and the two related species, *P. hanburyi* and *P. obtusifolium*. PCR with *PaB6*-specific primers yielded one strong band of approx. 250 bp, corresponding to the *PaB6* monomer (Fig. 2A), a second band of approx. 120–130 bp and, occasionally, a third band of approx. 60–80 bp (Fig. 2B). The main bands, corresponding to the expected size of the monomer of *PaB6*, were isolated, cloned and sequenced from two or three individuals of each of the six taxa/cytotypes. Amplification of dimers or even longer fragments was not observed, or observed very rarely.

The outgroup taxa of the family Hyacinthaceae were subjected to the same PCR amplification protocol and primers. Representatives of the related genera *Othocallis* and *Barnardia* showed no bands after PCR amplification, regardless of the annealing temperature (Fig. 2B). Very faint, monomer-related bands, close to the limit of detection, were seen occasionally, without any consistent pattern regarding annealing temperature or taxon, and were regarded as contamination (Fig. 2B, and not shown).

Sequence analysis of 66 cloned *PaB6* monomers (Supplementary Data Table S1), representing monomers amplified from two or three individuals of each of the six diploid taxa, confirmed that they all carried *PaB6* repeats. Fifty-one of these (83 %) were 249 bp long, with 12 shorter (18 %; 119, 175, 243, 247 and 248 bp) and three longer (4·5 %; 250 and 256 bp, the latter due to a TTAGGG insertion). High overall levels of sequence similarity amongst the amplified population of *PaB6* monomers, both within (93–100 %) and between (92–100 %) the different diploid cytotypes of *P. autumnale* and two other *Prospero* species, were observed (Supplementary Data Table S2). Thus, the intercytotype sequence variation of repeats amplified with the reconstructed monomer primers was as equally low and random as that within cytotypes or between individuals. The variation was mostly due to single base pair indels or point mutations occurring at different positions along the monomer, and these were monomer specific (alignment available upon request).

Neighbour-net analyses of DNA sequences of all cloned inserts of *PaB6* repeats from the six cytotypes corroborated the analyses of variation within the monomers, and did not reveal any cytotype-specific lineages (Fig. 2C). Instead, the repeats originating from different individuals were intermingled, regardless either of *PaB6* overall copy number and abundance or of their phylogenetic relationship.

### Copy number variation and genomic organization of *PaB6*

**Fig. 3.**
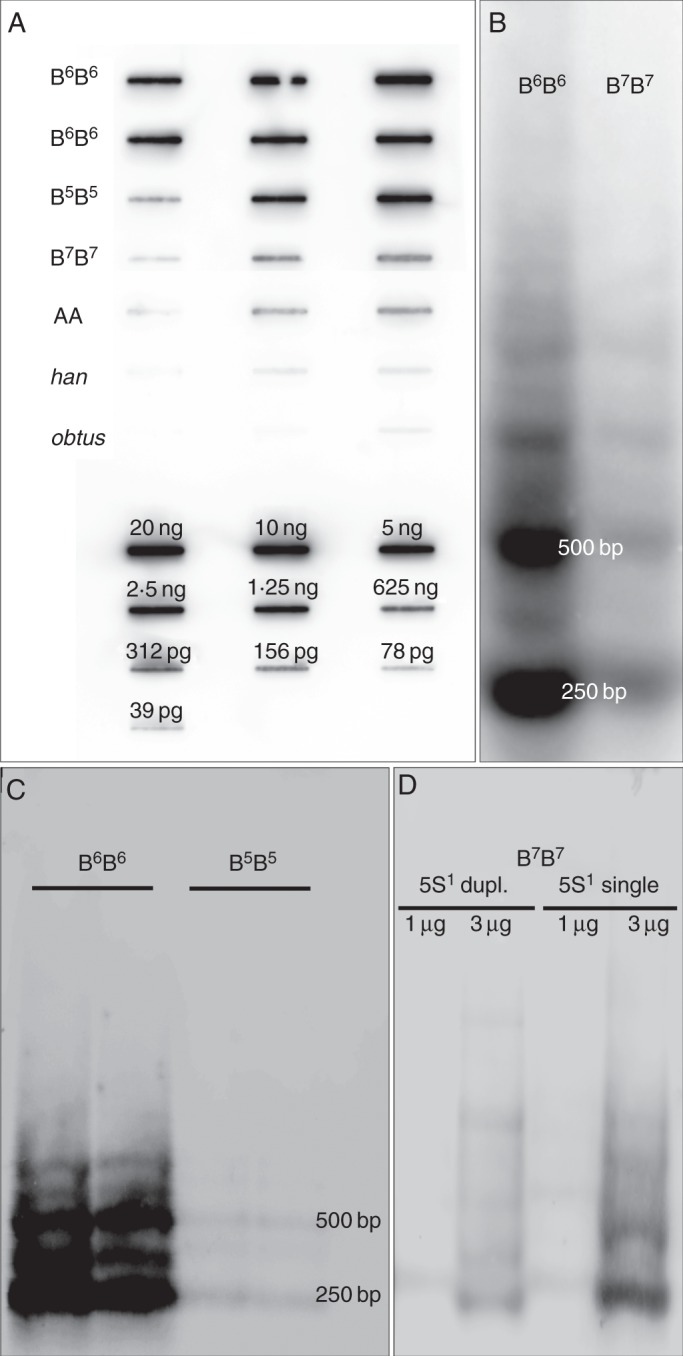
Copy number estimation of *PaB6* using slot blotting (A) and analyses of genomic organization of *PaB6* repeats in *Prospero* using (B–D) Southern blot hybridization. (A) Slot blot for *PaB6* copy number determination; DNA amount: 2, 20, 100 ng (for B^6^B^6^ and B^5^B^5^); 20, 200 and 2000 ng for B^7^B^7^ and AA; 200 and 2000 ng for *P. obtusifolium* (*obtus*) and *P. hanburyi* (*han*) (Supplementary Data Table S1). (B) Radioactive detection of digoxigenin-labelled probe *PaB6* hybridized to genomic DNA of cytotype B^6^B^6^ (H166) and B^7^B^7^ (H428). (C, D) Chemiluminescent detection of digoxigenin-labelled *PaB6* probe in restricted genomic DNA: (C) B^6^B^6^ (H166, H468) and B^5^B^5^ (H637, H565); (D) B^7^B^7^ (H424, duplicated 5S^1^ rDNA) and B^7^B^7^ (H428, single 5S^1^ rDNA) each with 1 and 3 μg of DNA.

**Fig. 4.**
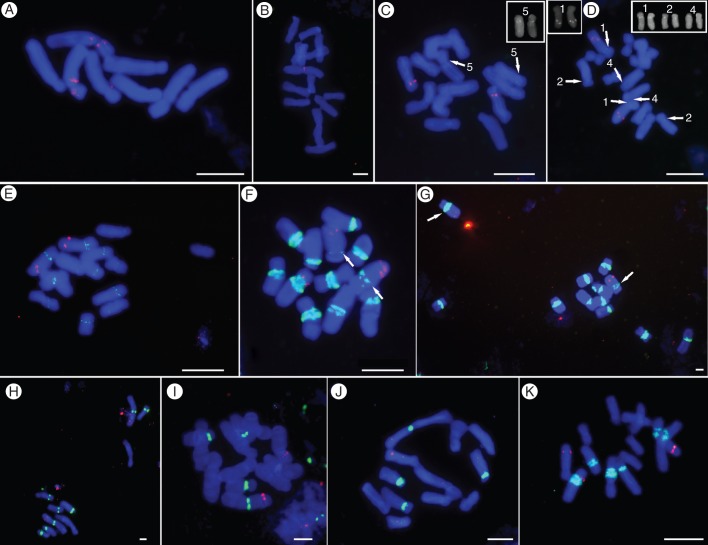
Localization of *PaB6* in chromosomes of diploid *Prospero* species and cytotypes, and in three homoploid hybrids. The *PaB6* loci are shown as green signals, and 5S rDNA in red. (A) *P. obtusifolium* (H563), (B) *P. hanburyi* (H115), (C**–**K) *P. autumnale* complex: (C) cytotype AA (H551, inset: chromosomes carrying *PaB6* signals), (D, E) B^7^B^7^ with duplicated (D: H424, left inset, duplicated 5S rDNA signals; right inset, chromosomes carrying *PaB6* signals) and single (E: H440) 5S rDNA locus in chromosome 1, (F, G) B^6^B^6^ with weak (F: H195) and strong (G: H427) signal of *PaB6* in chromosome 2 (arrows), (H) B^5^B^5^ (H581), (I) AB^5^ (H567), (J) B^5^B^7^ (H633), (K) B^6^B^7^ (H518) diploid hybrid. Each individual has a unique ID (in parentheses, e.g. H563; see Supplementary Data Table S1). Scale bar = 5 μm.

Copy numbers were estimated by quantitative chemiluminescent dot blot hybridization of labelled *PaB6* as probe against known quantities of genomic DNAs of all three species and four cytotypes (Fig. 3A). Large differences in the genomic content of *PaB6* between the four cytotypes of *P. autumnale* corroborated the results of Southern blot experiments and of FISH (Figs 3B–D and 4). The probe hybridized strongly to genomic DNA of cytotype B^6^B^6^ (Fig. 3A–C), moderately to cytotype B^5^B^5^ (Fig. 3A, C) and weakly to some individuals of cytotype B^7^B^7^ (Fig. 3A, B, D). A very weak signal was detected in genomic DNA of cytotype AA (Fig. 3A). The *PaB6* probe hybridized only very weakly to DNAs of *P. hanburyi* and *P. obtusifolium* (Fig. 3A). The highest copy number was found in cytotype B^6^B^6^ with 1·8–2·1 × 10^6^ copies per haploid genome (7–10 %), followed by cytotype B^5^B^5^ with 1·2–1·4 × 10^6^ copies/1C (6–7 %). One accession of cytotype B^7^B^7^ had 2·1–2·5 × 10^4^ copies/1C (approx. 0·13 %), while AA had 1·8–2·6 × 10^4^ copies/1C (approx. 0·08 %; Table 1, Fig. 3A). A variant of the B^7^B^7^ cytotype, carrying a single 5S rDNA locus on chromosome 1 and stronger signals of *PaB6* in all chromosomes, could not be analysed due to lack of appropriate quality plant material. *Prospero obtusifolium* and *P. hanburyi* possessed only very low amounts of *PaB6*, below the slot blot detection limit.

**Table 1.** Characterization of satellite *PaB6* repeats in diploid species and cytotypes of the genus *Prospero*

Taxon	2*n*	Genome proportion % of *PaB6*	Copy number/1C of *PaB6*	Genome size (pg) per 1C*	Figure
*P. autumnale*					
Cytotype B^5^B^5^	10	6·3–7·4	1·25–1·37 × 10^6^	4·86 ± 0·002	2A; 3A, C; 4H
Cytotype B^6^B^6^	12	7·16–10·71	1·76–2·06 × 10^6^	6·27 ± 0·083	2A; 3A–C; 4F, G; 5A, B
Cytotype B^7^B^7^: single 5S^1^ rDNA	14	NA	NA	4·23 ± 0·048	2A; 3B, D; 4E
Cytotype B^7^B^7^: duplicated 5S^1^ rDNA	14	0·12–0·14	2·11–2·49 × 10^4^	4·45 ± 0·023	2A; 3A, D; 4D
Cytotype AA	14	0·06–0·08	1·75–2·56 × 10^4^	7·85 ± 0·045	2A; 3A; 4C
*P. hanburyi*	14	ND	ND	6·81 ± 0·017	2A; 3A; 4B
*P. obtusifolium*	8	ND	ND	4·94 ± 0·039	2A; 3A; 4A

NA, not analysed due to lack of material; ND, copy number could not be determined due to very low *PaB6* contents.

*Jang *et al*. (2013).

Southern blot hybridization, using the satellite DNA single repeat (monomer) isolated from cytotype B^6^B^6^ as probe, was congruent in estimations of copy number of *PaB6* repeats and also enabled analysis of their genomic organization. The hybridization pattern of *PaB6* was typical of tandemly repeated DNAs, with the major 249 bp band and its multiples being most prominent in all samples. An additional, weaker, band about 375 bp in length, corresponding to an additional major sub-unit (Fig. 3B, C), has also been detected in all samples.

Methylation of *PaB6* repeats was analysed in cytotype B^6^B^6^, after digestion with methylation-insensitive (*Bst*NI) and methylation-sensitive (*Scr*FI) restriction enzymes with the same recognition site. The satellite DNA monomers were heavily methylated at CHG sites (Supplementary Data Fig. S3).

### Chromosomal localization and organization of *PaB6* repeats

*PaB6* has been localized in all six cytotypes using FISH (Supplementary Data Table S3).

The variation in number and size of satellite DNA loci detected corresponded well to the Southern slot results. Thus *P. obtusifolium* (Fig. 4A) and *P. hanburyi* (Fig. 4B) had no *PaB6* loci detectable by FISH due to very low copy numbers of *PaB6* monomers (Figs 3A and 4A–B). *Prospero autumnale* diploids, in contrast, all exhibited hybridization signals using FISH, but were variable in numbers of sites and in signal strengths (Fig. 4C–K). *PaB6* is predominantly located in pericentromeric regions of at least one, and sometimes all, chromosome pairs, and might, at least partly, span the centromeres (Supplementary Data Fig. S2).

In cytotype B^6^B^6^, major loci were present on all chromosomes of the complement (Fig. 4F, G). The pattern of satellite distribution was remarkably uniform between individuals and populations, and loci were of similar signal strength. Chromosome 1 showed the only polymorphism, with the locus size varying between homologues in some individuals (Fig. 4F, G).

In B^5^B^5^, *PaB6* loci occurred on four of the five chromosome pairs (Fig. 4H) and were of similar signal strength. Chromosome 3 showed, at most, a very weak hybridization signal (Supplementary Data Table S3, and data not shown).

Cytotype AA had only a single locus of *PaB6* – on chromosome 5 – but this was weak and barely detectable (Fig. 4C). The most variable *PaB6* distribution was shown by cytotype B^7^B^7^. Some individuals possessed medium-sized signals in pericentromeric regions of all chromosomes (Fig. 4E; Supplementary Data Table S3), while others had much weaker signals limited to three chromosome pairs (Fig. 4D; Supplementary Data Table S3). These patterns correlated with a duplication polymorphism of 5S rDNA present on chromosome 1 (5S^1^; see also Fig. 6). Thus the five individuals with a single 5S^1^ rDNA locus showed moderate amplification of *PaB6* on all chromosomes (Fig. 4E), while the six plants with a duplicated 5S locus carried weakly amplified *PaB6* loci only on chromosomes 1, 2 and 4 (Fig. 4D). The number and localization of *PaB6* satellite DNA loci in all cytotypes are shown in Fig. 6 and Supplementary Data Table S3.

All six F_1_ diploid hybrids possessed perfectly additive numbers and strengths of *PaB6* loci compared with their diploid parents. This was supported by Southern blot hybridization of a B^6^B^7^ hybrid, which also indicated additivity (not shown).

**Fig. 5.**
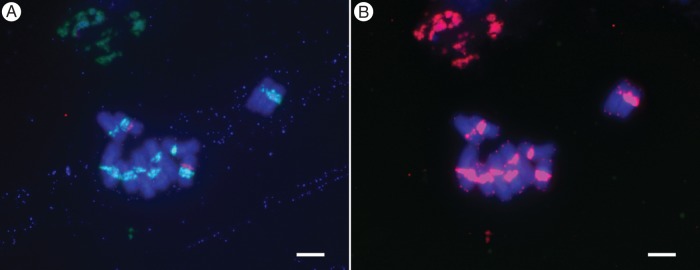
Localization of telomeric PNA probe (TTAGGG) and satellite *PaB6* in chromosomes of diploid *Prospero autumnale* cytotype B^6^B^6^ (H468): (A) *PaB6* loci (green) and 35S rDNA locus (red); (B) telomeric PNA probe (red) localized to the same metaphase chromosomes spread as in (A). Scale bar = 5 μm.

The *PaB6* monomer contains seven perfect and a few imperfect vertebrate-type telomeric sequences (TTAGGG), typical of the monocot order Asparagales to which *Prospero* belongs. TTAGGG sequences were detected at chromosome ends (Fig. 5) but additionally co-localized with the *PaB6* loci. Signal intensity in the pericentric chromosome regions using a telomeric DNA probe corresponded to signal strength and localization of the *PaB6* probe itself (Fig. 5A, B).

## DISCUSSION

Tandem repeats localize to heterochromatic segments in chromosomes (Hemleben *et al.*, 2007). *Prospero* cytotypes differ in the amount and distribution of heterochromatin, both among and within cytotypes. So far, only the cytotypes AA, B^7^B^7^ and B^6^B^6^ have been analysed using C-banding (Ebert *et al*., 1996) and the only consistently detectable heterochromatic blocks co-localized with nucleolar organizer regions (NORs). However, cytotype B^6^B^6^ had a high amount of heterochromatin, detected as blocks (C-bands) in the pericentric regions of all chromosomes (Ebert *et al.*, 1996). This was the rationale for selecting the B^6^ genome for repetitive DNA fraction analyses. Cytotype B^7^B^7^ was very variable in the number of heterochromatic blocks, but these were mainly dot-like and localized interstitially, except for slightly larger pericentric blocks which varied in size between individuals. Cytotype AA had only small interstitial heterochromatic blocks on six of the seven pairs. All of these pericentric heterochromatic blocks detected by Ebert *et al*. (1996) correspond to *PaB6* signals. The additional, smaller and more polymorphic interstitial bands detected are most likely to be composed of other tandem repeat(s), some of which might be cytotype specific.

Satellite DNA repeats represent a substantial proportion of the genomes of many higher plants (e.g. VicTR-A/B in *Vicia*, Macas *et al*., 2000; FriSAT1 in *Fritillaria*, Ambrožová *et al*., 2011). The *PaB6* repeat of *Prospero* is one of the most abundant satellites reported so far (Hemleben *et al*., 2007). It represents about 10 % of the genome in the B^6^B^6^ cytotype with 1·4 × 10^6^ copies. In comparison, tandem repeat VicTR-A/B comprises about 1 % of the genome of most *Vicia* species with 10^6^ copies (VicTR-A) but reaches 25 % of the genome with 1 × 10^6^–5 × 10^6^ copies (VicTR-B) in *V. sativa* (Macas *et al*., 2000), approaching the highest value reported in plants for the FokI element in *V. faba* (2·5 × 10^7^ copies/1C; Kato *et al*., 1984). The 37–55 bp long PAF1 repeat in *Picea abies* occurs in 2·7 × 10^6^ copies/1C (approx. 0·6 %; Sarri *et al*., 2008), while MCSAT in *M. comosum* has 9 × 10^5^ copies representing 5 % of the genome (de la Herrán *et al*., 2001).

*PaB6* is exceptional for its copy number variation between the closely related diploid cytotypes of one species complex. The satellite can clearly expand from a few hundred base pairs up to several hundred megabases in a relatively short evolutionary period. Such rapid changes should be reflected by genome size differences between *Prospero* cytotypes. The genome sizes of the derived cytotypes B^5^B^5^ and B^6^B^6^ are distinctly higher than those of cytotype B^7^B^7^, which has been inferred to be most similar to the ancestral karyotype (Jang *et al*., 2013; K. Emadzade *et al*., unpubl. res.). *PaB6* amplification significantly contributes to these genome size increases and gives rise to heterochromatic blocks in B^6^B^6^. The correlation between genome size and *PaB6* amount is particularly evident in the comparison of the youngest cytotype B^5^B^5^ and its close relative, and likely ancestor, B^7^B^7^ (Jang *et al*., 2013). The B^5^ genome is about 400 Mb (10 %) larger than the B^7^ genome, half of which can be attributed to *PaB6* copy number increase (325 Mb in B^5^B^5^ vs. 7 Mb in B^7^B^7^). In contrast, the large size of the A genome is clearly not associated with the high copy number of *PaB6*.

Satellite DNA copy number can change relatively rapidly due to expansions and contractions of satellite arrays. Thus, the copy number of FRISAT1 in the genus *Fritillaria* varies within and between different subgenera (Ambrožová *et al*., 2011), and several genus-specific satellite DNAs differ in copy numbers between related *Secale* (Cuadrado and Jouve, 2002) and *Nicotiana* species (Lim *et al*., 2004). Such differences are also observed between varieties and cultivars of *Phaseolus vulgaris* and maize (Peacock *et al*., 1981; Ribeiro *et al*., 2011) indicating the highly dynamic character of satellite repeats. These changes may be accompanied by divergence of the monomer sequences during evolution, via accumulation and fixation of mutations in satellite families (Plohl *et al*., 2008). Interestingly, in *Prospero*, despite the dynamic changes in copy number, there is no indication of sequence divergence during lineage evolution.

In *Barnardia* and *Othocallis* (Fig. 2B), genera closely related to *Prospero* (Pfosser and Speta, 1999; Ali *et al*., 2012), no *PaB6* monomers were detected, shown by a lack of amplification of *PaB6* monomer-equivalent bands in PCR. Thus, *PaB6* probably evolved during the emergence of the genus *Prospero*, and remained in low copy number as part of the library of repeats (Meštrovič *et al*., 1998) in the chromosomally stable species *P. obtusifolium* and *P. hanburyi. PaB6* amplification, therefore, is specific to the chromosomally dynamic *P. autumnale* complex.

*PaB6* dynamics can be assessed against the phylogeny of the genus (Jang *et al*., 2013). *Prospero obtusifolium* and *P. hanburyi* possess very few *PaB6* monomers, and these can only be detected by PCR, because they are below the detection limit of all types of *in situ* hybridization. In contrast, the four diploid cytotypes of *P. autumnale* all possess *PaB6* in amounts detectable by FISH and genomic DNA hybridization, although copy number varies substantially. *PaB6* in B^6^B^6^ represents 8–10 % of the genome and 6–7 % in B^5^B^5^. Copy number estimation from NGS data, however, suggests that *PaB6* represents about 1·8 % of the B^6^B^6^ genome, only a quarter of that from slot blot hybridization. This discrepancy is probably caused by *PaB6* under-representation due to a bias affecting template preparation from satellite repeats during 454 sequencing (Macas *et al*., 2007; J. Macas *et al*., unpubl. res.).

**Fig. 6.**
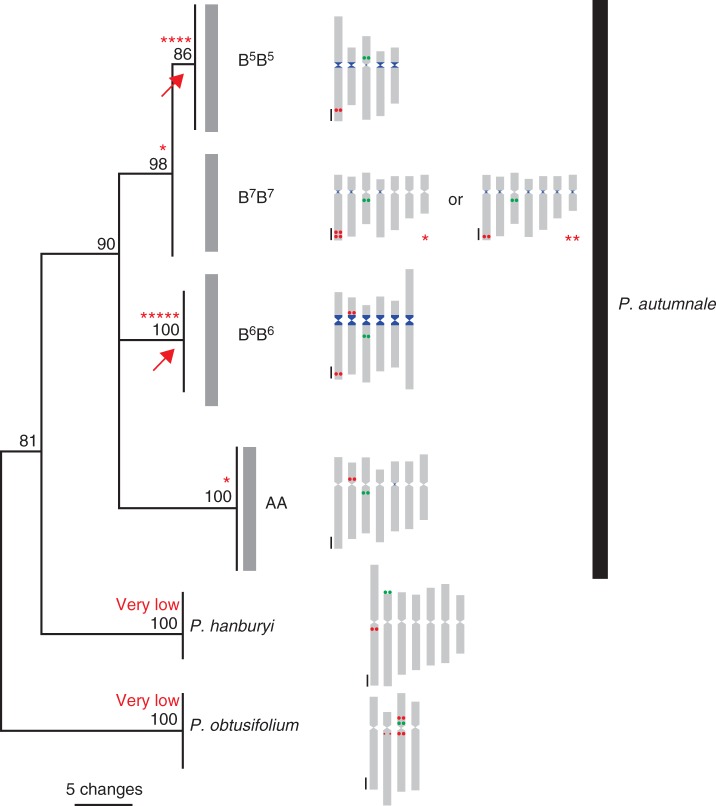
Model of evolution of *PaB6* in diploid taxa of *Prospero.* Idiograms of all analysed species and cytotypes are mapped onto the ITS (internal transcribed spacer) tree (adapted from Jang *et al*., 2013). *PaB6* satellite DNA is indicated as blue blocks, 5S rDNA as red circles and 35S rDNA as green circles. Asterisks indicate lineages which have experienced significant amplification of *PaB6*. Arrows mark amplification events accompanying fusions.

In some plant and animal groups, patterns of copy number variation of a satellite DNA family in a group of closely related taxa carry a phylogenetic signal. However, similarity in copy number might result from independent satellite amplifications or contractions (Rosato *et al*., 2012). The two *Prospero* cytotypes whose genomes are enriched in *PaB6* have reduced basic chromosome numbers of *x* = 6 and *x* = 5 derived from *x* = 7 via independent fusion events, so do not demonstrate a sister relationship (Jang *et al*., 2013; Fig. 6). Thus, the raised amounts of *PaB6* in these two cytotypes could have resulted from independent amplifications, coinciding with fusions leading to basic number changes. This is particularly plausible for the phylogenetically young cytotype B^5^B^5^, which is nested within B^7^B^7^, a cytotype carrying relatively few copies of *PaB6* (Jang *et al*., 2013; Fig. 6). However, high copy numbers in these two unrelated lineages might be a remnant of a common amplification event which was followed by differential loss. This hypothesis is more plausible for cytotype B^6^B^6^ than for B^5^B^5^. B^6^B^6^ clearly originated from *x* = 7, but does not strongly relate, phylogenetically or chromosomally, to any lineage of present-day B^7^B^7^, and may have arisen directly from the ancestral cytotype, or an as yet undiscovered B^7^ lineage, with high copy numbers of *PaB6* (Jang *et al*., 2013). Thus, the lack of phylogenetic evidence of copy number of *PaB6* in the ancestral karyotype of *Prospero* leaves the question open.

The presence of telomeric motifs in the *PaB6* sequence is interesting with respect to the high karyotype instability within and between all *P. autumnale* cytotypes (Vaughan *et al.*, 1997; Jang *et al.*, 2013). The presence of interstitial telomeric repeats (ITRs) is often interpreted as a remnant of evolution by telomere–telomere chromosomal fusions. However, it may also result from rearrangements such as translocations or inversions (Uchida *et al*., 2002; Ruiz-Herrera *et al*., 2008; Rosato *et al*., 2012), particularly whole chromosomal arm inversions involving both the centromere and telomere (Presting *et al*., 1996). The occurrence of telomeric repeats within, or at the margins of, constitutive heterochromatin has been reported in vertebrates (Meyne *et al*., 1990) but is also known in plants (Presting *et al*., 1996; Uchida *et al*., 2002; Weiss-Schneeweiss *et al*., 2004; Mlinarec *et al.*, 2009; Gong *et al*., 2012; He *et al*., 2013). It has been argued that these telomeric repeats can be an integral and long-established part of the satellite DNAs of constitutive heterochromatin (Slijepcevic *et al*., 1996; Garrido-Ramos *et al*., 1998; Metcalfe *et al*., 2004), originally inserted and amplified through DNA double strand breaks (DSBs) repaired by telomerase (Nergadze *et al*., 2004, 2007). The ITRs detected in *Prospero* are certainly an integral part of *PaB6* interspersed amongst other sequence motifs. Their origin, however, cannot be unambiguously established.

Two mechanisms have been proposed for satellite DNA copy number change: unequal crossing-over with gene conversion (Liao, 1999; Eickbush and Eickbush, 2007), and amplification and homogenization of monomers by extrachromosomal circular DNA (eccDNA, ‘rolling circle’) molecules during recombination (Navrátilová *et al*., 2008; Cohen *et al*., 2010). They are not mutually exclusive and might operate in concert, resulting in mobility and homogenization of repetitive DNAs. Whether these mechanisms are also involved in expansion of *PaB6* in *Prospero* remains unknown.

Although copy number varies hugely between cytotypes within *Prospero*, the monomer sequence is conserved. This may indicate either relatively recent amplification of the monomer or efficient systems of sequence homogenization and gene flow between taxa (Hemleben *et al*., 2007). The geographically disjunct distributions of the cytotypes AA, B^5^B^5^ and B^6^B^6^, and consequent lack of gene flow between them, suggest that *PaB6* represents a recent and highly dynamic system originating from a small pool of ancestral repeats (Mravinac *et al*., 2005; Plohl *et al*., 2010).

## SUPPLEMENTARY DATA

Supplementary data are available online at www.aob.oxfordjournals.org and consist of the following. Figure S1: NGS graph layouts and dot-plots of the contigs. Figure S2: pericentric localization of *Pa*B6 in cytotype B^6^B^6^. Figure S3: methylation levels of *PaB6*. Table S1: plants used and collection details, GenBank accession numbers (*PaB6*), and methods used for analysis. Table S2: sequence similarity of cloned monomers of *PaB6* satellite DNA within and between different diploid cytotypes of *Prospero*. Table S3: characterization of 5S rDNA and satellite DNA *PaB6* loci in chromosomes of diploid species, cytotypes and hybrids of the genus *Prospero*.

Supplementary Data
