# Supplementary material for: Differential amplification of satellite PaB6 in chromosomally hypervariable Prospero autumnale complex (Hyacinthaceae)
Source: Ann Bot. 2014 Aug 28;114(8):1597–608. doi: 10.1093/aob/mcu178 (PMC4273535; doi:10.1093/aob/mcu178)
Supplement: Supplementary Data [file supp_114_8_1597__index.html]

Differential amplification of satellite PaB6 in chromosomally hypervariable Prospero autumnale complex (Hyacinthaceae) — Differential amplification of satellite PaB6 in chromosomally hypervariable Prospero autumnale complex (Hyacinthaceae) — Supplementary Data 

# Differential amplification of satellite *PaB6* in chromosomally hypervariable *Prospero autumnale* complex (Hyacinthaceae)

## Supplementary Data

Supplementary Data

**Files in this Data Supplement:**

- Supplementary Data - Pdf file
