# Supplementary material for: Differential amplification of satellite PaB6 in chromosomally hypervariable Prospero autumnale complex (Hyacinthaceae)
Source: Ann Bot. 2014 Aug 28;114(8):1597–608. doi: 10.1093/aob/mcu178 (PMC4273535; doi:10.1093/aob/mcu178)
Supplement: Supplementary Data [file supp_mcu178_mcu178supp.pdf]

SUPPLEMENTARY DATA

Fig. S1. Graph layout (cluster CL0009) identified by NGS, and dot-plot of the sequences of longest contigs of cluster 9 indicating the presence of tandem repeats in head-to-tail orientation.

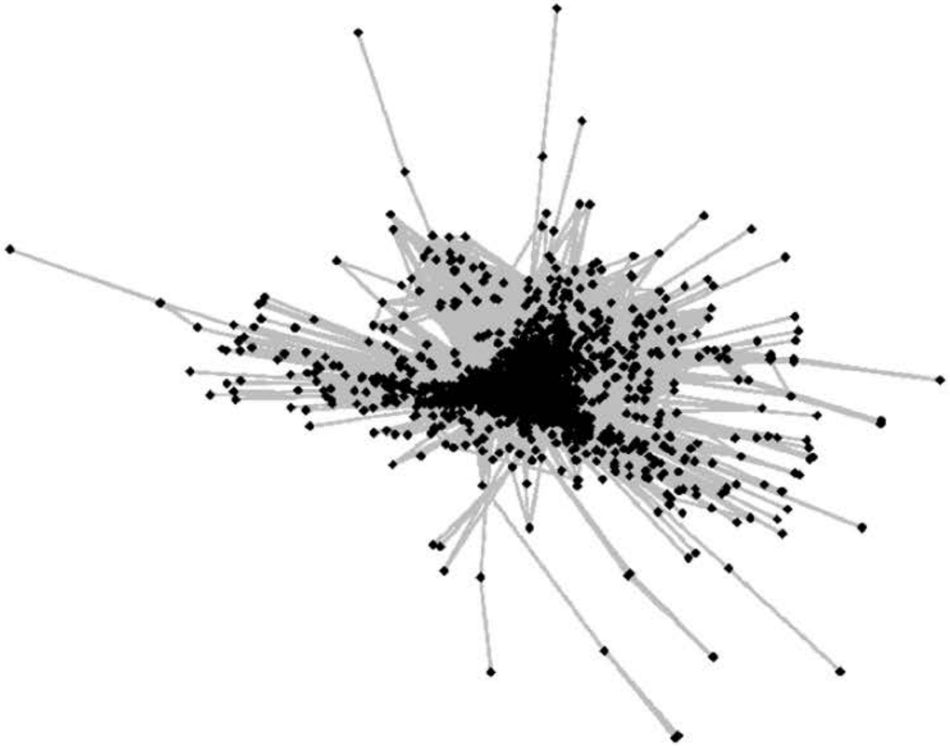

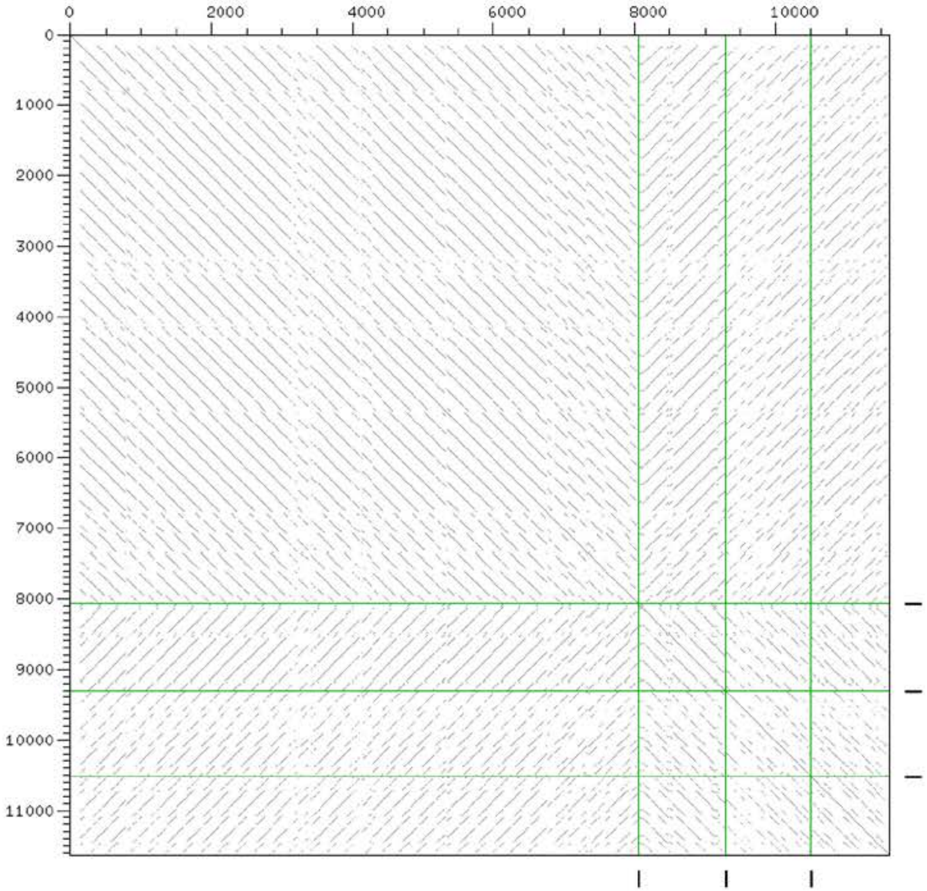

Fig. S2. Distribution of *PaB6* in pericentric and centromeric regions on all chromosomes in cytotype B^6B^6. Arrow indicates *PaB6* signal in extended centromeric region.

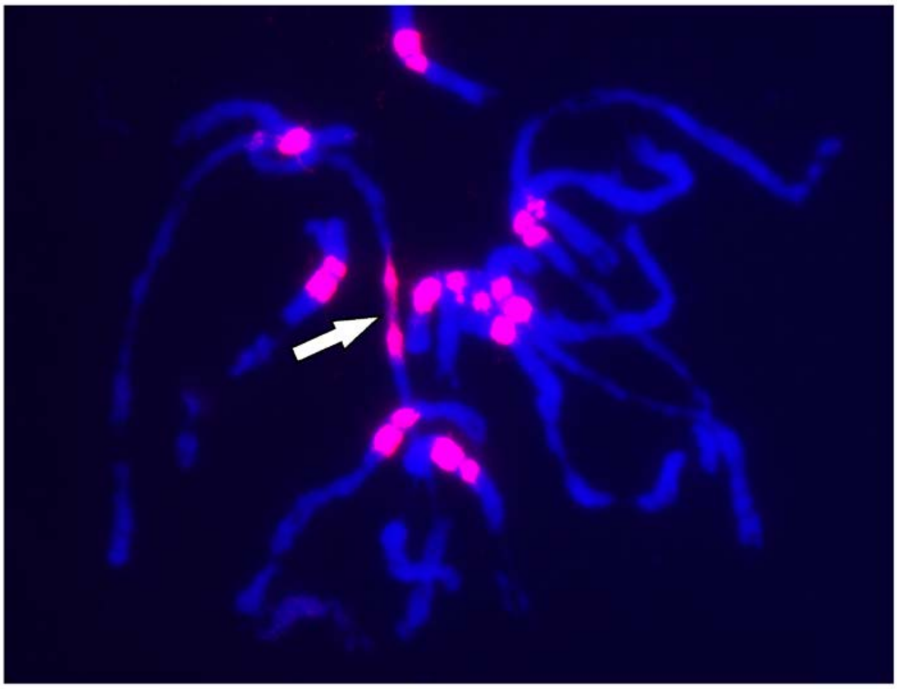

Fig. S3. *PaB6* in the B^6 genome (H247) restricted with *Bst*NI and *Scr*F1, showing high cytosine methylation of satellite monomers, particularly at CHG motifs.

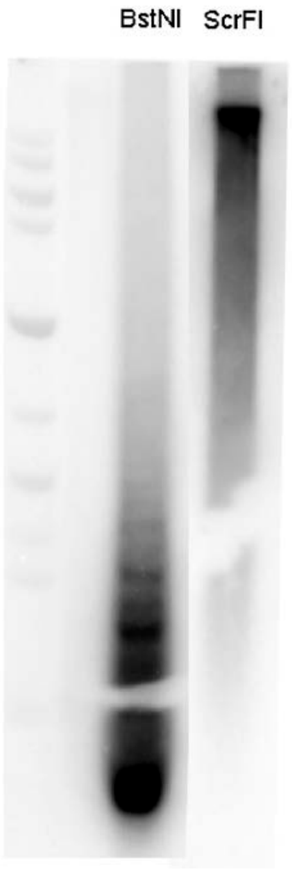

Table S1. Plants used and collection details, GenBank accession numbers (*PaB6*), and methods used for analysis. All plants cultivated in HBV (Vienna, Austria).

Taxon (chromosome number, cytotype)	Locality; Collection; accession number	Methods applied to analyze *PaB6*	GenBank accession numbers of *PaB6* fragments
P. hanburyi (2*n* = 14)	Turkey, Findikpinar; Speta; H115	PCR, Seq, SB, FISH	KF897635-KF897639
	Turkey, Findikpinar; Speta; H397	PCR, Seq, SB, FISH[1]	KF897640-KF897642
	Turkey, Narlikuyu, Silifke; H231	FISH, CNE	---
P. obtusifolium (2*n* = 8)	Morocco; Parker; H547	Seq, SB	KF897643-KF897647
	Morocco; Parker; H563	PCR, FISH	---
	Morocco; Parker; H564	FISH	---
	Spain; Parker; H559	PCR, Seq, SB	KF897648-KF897652
	Spain; Parker; H540	SB, FISH[1], CNE	---
P. autumnale			
(2*n* = 14, AA)	Spain, Huelva; Parker; H541	PCR, Seq, SB	KF897587-KF897591
	Spain, Badajoz; Parker; H543	Seq, SB, FISH[1]	KF897592-KF897596
	Spain, Jaen; Parker; H546	SB	---
	Spain, Huelva; Parker; H548	SB, FISH, CNE	---
	Portugal, Peniche; Parker; H550	PCR, SB, FISH	---
	Portugal, Peniche; Parker; H551	FISH	---
	Portugal, Peniche; Parker; H552	SB	---
(2*n* = 10, B^5B^5)	Libya, Tobi; Parker; H566	Seq, SB, CNE	KF897597-KF897601
	Libya, Tobi; Parker; H581	FISH	---
	Libya, Tobi; Parker; H582	PCR, Seq, SB	KF897602-KF897606
	Libya, Nagasa; Parker; H640	PCR, FISH	---
(2*n* = 12, B^6B^6)	Greece, Crete; Speta; H166	PCR, Seq, SB	KF897612-KF897616
	Greece, Crete; Speta; H170	SB, FISH	---
	Greece, Crete; Speta; H195	Seq, SB, FISH, CNE	KF897617-KF897620
	Greece, Crete; Speta; H271	SB	---
	Greece, Crete; Speta; H274	SB, FISH	---
	Greece, Crete; Speta; H427	PCR, Seq, SB, FISH, CNE	KF897607-KF897611
	Greece, Crete; Speta; H455	SB	---
	Greece, Crete; Speta; H468	FISH[1]	---
(2*n* = 14, B^7B^7)	Greece, Crete; Speta; H47	SB, FISH[3]	---
	Greece, Crete; Speta; H58	Seq, SB	KF897630, KF897632-KF897634
	Greece, Crete; Speta; H89	Seq, SB	KF897625-KF897629
	Greece, Crete; Speta; H174	SB	---
	Greece, Crete; Speta; H440	SB, FISH[1,3]	---
	Greece, Crete; Speta; H464	SB	---
	Greece, Lefkas, Nikiana; Speta; H432	SB	---
	Greece, Kalamitsi; Speta; H450	SB, FISH[3]	---
	Greece, Poros; Speta; H535	Seq, SB	KF897621-KF897624
	Greece, Rhodes; Speta; H137	FISH[2]	---
	Israel, Nene Han; Parker; H612	FISH[3]	---

	Italy, Sicily; Speta; H428	PCR, SB, FISH[3]	---
	Italy, Sicily; Speta; H403	SB	---
	Montenegro; Speta; H422	FISH[1,2]	---
	Montenegro; Speta; H424	PCR, SB, FISH[1,2], CNE	---
	Montenegro; Speta; H430	SB	---
	Serbia, Siget-Baun; Rat; H576	FISH[2]	---
	Turkey; Speta; H179	SB	---
	Ukraine, Nikita; Kish; H591	FISH[2]	---
	Speta; H228	FISH[2]	---
	Speta; H433	SB	---
Diploid homoploid hybrids			
($2n = 12$, AB^5)	Cross pollination experiment; Parker; H567	SB, FISH[1]	---
($2n = 12$, B^5B^7)	Cross pollination experiment; Parker; H633	FISH	---
($2n = 13$, B^6B^7)	Greece, Mykonos; Speta; H294	SB	---
	Greece, Crete; Speta; H211	FISH[1]	---
	Greece, Crete; Speta; H518	FISH	---
	Greece, Crete; Speta; H700	FISH	---
Outgroups			
Barnardia scilloides	Cult. HBV; Jang 1	PCR	---
Othocallis mischtschenkoana	Cult. HBV; Li 778	PCR	---
Othocallis siberica	Cult. HBV; Hyac2159/1	PCR	---

Seq, Sequencing; SB, Southern blot; DB, Dot blot; FISH, Fluorescence *in situ* hybridization with 5S rDNA and *PaB6*; CNE, copy number estimation via slot blot; [1], FISH with PNA telomeric probe $(CCCTAA)_3$ and *PaB6*; [2]duplicated and [3]single locus of 5S rDNA in chromosome 1.

Table S2. Sequence similarity (%) of cloned monomers of *PaB6* satellite DNA within and between different diploid cytotypes of *Prospero* given as: (minimum), average ± s.d., (maximum).[1]

	P. autumnale B^7B^7	*P. autumnale* B^6B^6	*P. autumnale* B^5B^5	*P. autumnale* AA	*P. hanburyi*	*P. obtusifolium*
B^7B^7	(95) 98.4 ± 1.2 (100)					
B^6B^6	(94) 98.0 ± 1.3 (100)	(95) 96.5 ± 0.9 (99)				
B^5B^5	(92) 97.5 ± 1.6 (100)	(94) 96.6 ± 1.1 (99)	(94) 96.5 ± 1.2 (99)			
AA	(95) 98.3 ± 1.1 (100)	(94) 96.8 ± 1.2 (100)	(94) 96.5 ± 1.3 (100)	(94) 96.4 ± 1.5 (100)		
P. hanburyi	(94) 97.8 ± 1.5 (100)	(94) 96.2 ± 1.0 (100)	(94) 96.1 ± 1.2 (100)	(94) 96.5 ± 1.3 (100)	(95) 96.0 ± 1.3 (100)	
P. obtusifolium	(95) 98.1 ± 1.2 (100)	(93) 96.4 ± 1.2 (100)	(93) 96.5 ± 1.4 (100)	(93) 96.6 ± 1.6 (100)	(93) 96.2 ± 1.3 (100)	(93) 96.4 ± 1.5 (100)

[1]Comparison of all vs. all: (92) 97.55 ±1.5 (100).

Table S3. Characterization of 5S rDNA and satellite DNA *PaB6* loci in chromosomes of diploid species, cytotypes and hybrids of the genus *Prospero*.

Cytotype	Number and location of 5S rDNA loci	Number and location of *PaB6* loci	Signal strength
Prospero obtusifolium			
(2*n* = 8)	3: L-P^{2}, L-P^{3}, S-P^{3}	–	–
P. hanburyi			
(2*n* = 14)	1: L-P^{1}	–	–
P. autumnale complex			
AA (2*n* = 14)	1: S-P^{2}	1: S-P^{5}	Very weak
B^{7}B^{7} (2*n* = 14)	1: L-D^{1**}	3: S-P$^{1, 2, 4}$	+
	1: L-D^{1*}	6: S-P$^{1, 2, 4, 5, 6, 7}$	++
B^{6}B^{6} (2*n* = 12)	2: L-D^{1}, S-P^{2}	6: S-P$^{1\text{-hetero}, 2, 3, 4, 5, 6}$	+++++
	2: L-D^{1}, S-P^{2}	6: S-P$^{1, 2, 3, 4, 5, 6}$	+++++
B^{5}B^{5} (2*n* = 10)	1: L–D^{1}	4: S-P$^{1, 2, 4, 5}$	+++
	1: L–D^{1}	5: S-P$^{1, 2, 3\text{-hetero}, 4, 5}$	+++
Diploid homoploid hybrids			
AB^{5} (2*n* = 12)	1 (A): S-P^{2}; 1(B^{5}): L–D^{1}	4 (B^{5}): S-P$^{1, 2, 4, 5}$	–;++++
B^{5}B^{7} (2*n* = 12)	1 (B^{5}): L–D^{1}; 1 (B^{7}): L-D^{1*}	4 (B^{5}): S-P$^{1, 2, 4, 5}$;<br>6 (B^{7}): S-P$^{1, 2, 3, 4, 5, 6}$	++++;+
B^{6}B^{7} (2*n* = 13)	2 (B^{6}): L-D^{1}, S-P^{2}; 1 (B^{7}): L-D^{1*}	6 (B^{6}): S-P$^{1, 2, 3, 4, 5, 6}$	+++++; –

Note: S, short arm; L, long arm; P, pericentric region; D, distal region; $^{1\text{-}7}$, number of the chromosome; **, duplicated 5S^{1} rDNA signal; *, single 5S^{1} rDNA signal; +, ++, +++ etc., strength of *PaB6* signals; – no *PaB6* signals.
